# Near‐Room‐Temperature Magnetoelectric Coupling via Spin Crossover in an Iron(II) Complex

**DOI:** 10.1002/anie.202214335

**Published:** 2022-11-23

**Authors:** Magdalena Owczarek, Minseong Lee, Shuanglong Liu, Ella R. Blake, Chloe S. Taylor, Georgia A. Newman, James C. Eckert, Juan H. Leal, Troy A. Semelsberger, Hai‐Ping Cheng, Wanyi Nie, Vivien S. Zapf

**Affiliations:** ^1^ Center for Integrated Nanotechnologies Los Alamos National Laboratory Los Alamos NM 87545 USA; ^2^ National High Magnetic Field Laboratory Los Alamos National Laboratory Los Alamos NM 87545 USA; ^3^ Department of Physics Quantum Theory Project Center for Molecular Magnetic Quantum Materials University of Florida Gainesville FL 32611 USA; ^4^ Harvey Mudd College Claremont CA 91711 USA; ^5^ Materials Physics and Applications Division Los Alamos National Laboratory Los Alamos NM 87545 USA

**Keywords:** Magnetic Properties, Magnetoelectric Coupling, Molecular Magnet, Spin Crossover, Structural Phase Transition

## Abstract

Magnetoelectric coupling is achieved near room temperature in a spin crossover Fe^II^ molecule‐based compound, **[Fe(1bpp)_2_](BF_4_)_2_**. Large atomic displacements resulting from Jahn–Teller distortions induce a change in the molecule dipole moment when switching between high‐spin and low‐spin states leading to a step‐wise change in the electric polarization and dielectric constant. For temperatures in the region of bistability, the changes in magnetic and electrical properties are induced with a remarkably low magnetic field of 3 T. This result represents a successful expansion of magnetoelectric spin crossovers towards ambient conditions. Moreover, the observed 0.3–0.4 mC m^−2^ changes in the *H*‐induced electric polarization suggest that the high strength of the coupling obtained via this route is accessible not just at cryogenic temperatures but also near room temperature, a feature that is especially appealing in the light of practical applications.

## Graphical abstract

Magnetoelectric coupling is achieved near room temperature in a spin crossover Fe^II^ molecule‐based compound. The coupling is realized through the interplay of structural, magnetic, and electrical changes that allows the spin crossover to be driven by a remarkably low magnetic field of 3 T.

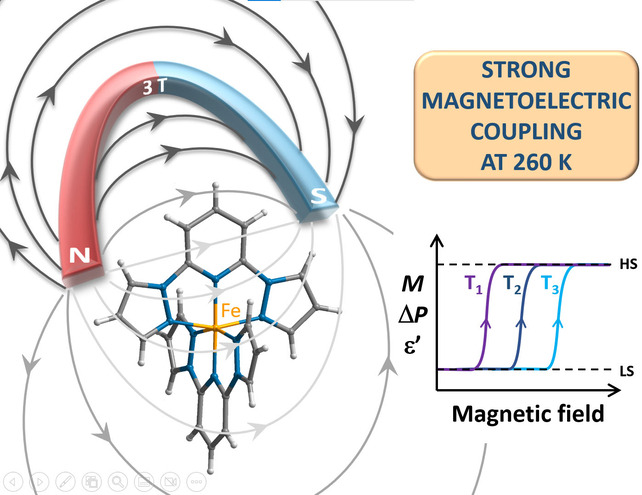

## Introduction

**Figure 1:**
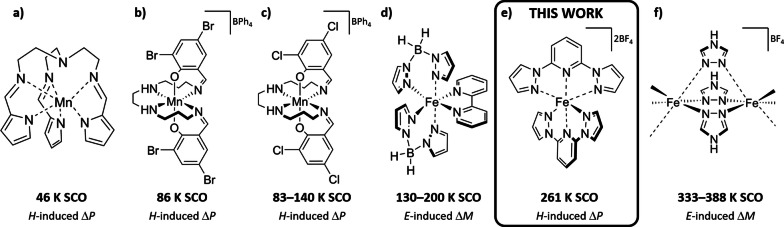
Magnetoelectric SCO molecule‐based compounds: a) Mn(taa), b) [Mn(3,5‐diBr‐sal)_2_323]BPh_4_, c) [Mn(3,5‐diCl‐sal)_2_323]BPh_4_, d) [Fe(H_2_B(pz)_2_)_2_(bipy)], e) [Fe(1bpp)_2_](BF_4_)_2_, f) [Fe(Htrz)_2_(trz)](BF_4_).

Molecule‐based complexes with 3*d* ^4^–3*d* ^7^ transition metal ions often undergo a spin crossover (SCO) between *d*‐orbitals of the ion that changes the magnitude of its total spin.[^1^, ^2^] The process is followed by large changes of the lattice parameters that, in turn, cause sharp and hysteretic changes in materials’ properties, e.g., structural, dielectric, optical, and mechanical properties. External parameters including pressure, magnetic field, electric field, light irradiation, and chemical adsorption[^3^, ^4^, ^5^, ^6^, ^7^, ^8^, ^9^, ^10^] can induce and influence SCOs at temperatures ranging from 50 to 400 K, providing many routes to multifunctional cross‐coupling of different properties. Using this opportunity, a new field of magnetoelectric (ME) coupling in molecule‐based SCO materials has recently emerged where magnetic field *H* influences the electric polarization *P* and dielectric constant and/or the electric field *E* influences the magnetization *M*.[^11^, ^12^, ^13^, ^14^, ^15^, ^16^, ^17^, ^18^] The coupling can be used in a numerous of technological applications, such as magnetic sensors, tunable antennas, energy harvesting, computing, and data storage[^19^, ^20^, ^21^, ^22^, ^23^, ^24^, ^25^, ^26^] with the important advantage that in these insulating materials the large power dissipation from electric currents in conducting materials is eliminated.[^19^, ^22^, ^27^, ^28^] In contrast to inorganic materials, the sub‐field of ME coupling in molecular materials and SCO is relatively new. As of now, a few compounds showing ME coupling have been reported with SCO temperatures either below 200 K or above 330 K (Figure 1) leaving the most desirable room temperature range still to be populated.

Strong ME coupling in SCO materials manifested as a strong coupling of the electric polarization and the magnetization, can be achieved when the lattice change at the transition involves at least one polar phase.[^11^, ^13^, ^15^, ^16^, ^29^, ^30^, ^31^] In our search for a suitable candidate to extend the family of SCO materials with ME coupling, we coupled this requirement with SCO transition occurring close to room temperature. As a result, we identified **[Fe(1bpp)_2_](BF_4_)_2_** (where 1bbp is 2,6‐di(pyrazol‐1‐yl)pyridine; Figure 1e) exhibiting SCO at ≈261 K as a good starting point of our investigation. Since the discovery of the compound's SCO properties in 2001,^[32]^ extensive studies of **[Fe(1bpp)_2_](BF_4_)_2_** have revealed other interesting properties, such as photomagnetic properties,[^33^, ^34^] barocaloric effect,^[35]^ and luminescence,^[36]^ and the fabrication of **[Fe(1bpp)_2_](BF_4_)_2_**‐based light‐emitting devices has also been reported.^[37]^ However, magnetoelectric coupling in this compound has not yet been investigated. In this work, we show that despite the fact that the SCO transition is isostructural, the molecular distortion at the SCO in **[Fe(1bpp)_2_](BF_4_)_2_** gives rise to strong changes in dielectric constant and electrical polarization. We observe these both as a function of temperature around 260 K, and as a function of magnetic fields as low as 3 T, evidencing ME coupling.

## Results and Discussion

**Figure 2:**
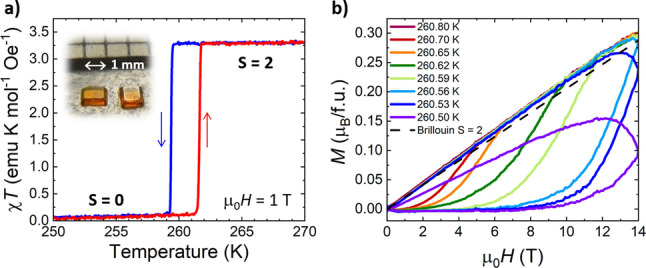
a) χ*T* vs. *T* measured at 1 T in a vibrating sample magnetometer (VSM) at 0.25 K min^−1^ on warming and cooling of a single crystal. Inset: a photograph of the as‐grown crystals. b) Magnetization (*M*) vs magnetic field (μ_0_ *H*) measured with a sweep rate of 50 Oe/s at different temperatures between 260.50–260.80 K for a single‐crystalline sample. A dashed line indicates the theoretical Brillouin function for the S=2 state.

**[Fe(1bpp)_2_](BF_4_)_2_** undergoes a hysteretic thermal spin crossover (SCO) centered around 260–261 K with a narrow hysteresis of approx. 2 K. The transition is represented as a sharp change in the magnetic susceptibility, plotted as χ*T* vs *T* in Figure 2a. The high temperature χ*T* value of 3.3 emu K mol^−1^ Oe^−1^ is consistent with the spin‐only value for a Fe^2+^ (d^6^) S=2, high spin (HS) state. An abrupt decrease of χ*T* value to ≤0.1 emu K mol^−1^ Oe^−1^ indicates a full conversion to S=0, low spin (LS) state. The presence of the first‐order transition centered around 261 K was confirmed by Differential Scanning Calorimetry (DSC, Figure S1).

The examination of the transition under magnetic fields revealed that the change in the total spin state can be also induced by magnetic fields. Figure 2b shows *M*(*H*) curves collected in a 14 T superconducting magnet on an unglued single crystal secured in a VSM powder holder so that B||*c*‐axis (perpendicular to the large face of the crystal). The crystal was cooled down to 240 K and slowly warmed up (0.3 K min^−1^) to the final measurement temperature and stabilized for additional 20 min. The magnetic sweep rate used was 50 Oe s^−1^. In a temperature region that is in the close vicinity of *T*_c_, 260.50–260.70 K, we observe a transition at a critical field with *M* close to zero at lower fields, consistent with the non‐magnetic state S=0, and quasi‐linear behavior at higher fields consistent with S=2 (g=2; dashed line in Figure 2b) confirming a full conversion (except for 260.50 K) of the molecule's LS state to its HS state. In this range of temperatures and fields, the magnetization of the Brillouin function expected from the S=2 paramagnetic HS state is roughly linear in *H*. As would be expected from energetic considerations, the closer the thermal SCO is approached from below in temperature, the lower the magnetic field that is needed to initiate field‐induced switching. For **[Fe(1bpp)_2_](BF_4_)_2_**, the lowest field needed to initiate the transition is 1 T at 260.7 K with 3.5 T to reach 50 % HS/LS. Notably, the field‐induced transition proceeds less sharply than the temperature‐induced transition change in χ*T*(*T*). The shape of the curves for 260.5 and 260.53 K indicate that the transition is so slow that it is continuing even after the magnetic field turns around. It is probable that the slower switching is related to the strength of the elastic interactions that are coupled to the SCO transition. This hypothesis seems to explain even slower field‐induced transition observed in *M*(*H*) curves obtained for a single crystal attached to a quartz rod with GE varnish (Figure S3). Being a first‐order phase transition, the transition proceeds by nucleation and phase growth, with the speed of the transition determined by the speed of propagation of the phase boundaries in the sample between the old and the new phase.

**Figure 3:**
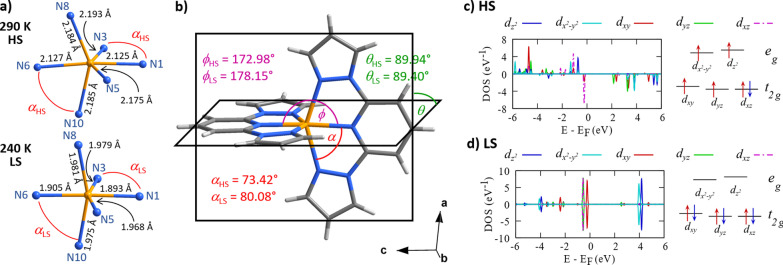
a) The coordination sphere of Fe^2+^ atom illustrating the change in Fe−N bond lengths due to Jahn–Teller distortion. b) The comparison of α, θ, φ angles (see text for the explanation) depicting the angular distortion adopted by **[Fe(1bpp)_2_]^2+^** cation in high (HS) and low (LS) spin states. The molecule in (b) is shown is its HS state. c) and d) PDOS for Fe *d* orbitals when the complex is in the high‐spin and low‐spin state, respectively, along with the simplified energy levels of *d*‐orbital and their occupations.

From the structural point of view, the SCO is accompanied by an isostructural transition leading the molecules in both LS and HS states to adopt a polar *P*2_1_ space group despite a Jahn–Teller distortion of the high‐spin complex and gradual freezing of dynamically disordered BF_4_^−^ anions at lower temperatures. The distortion of the cation in the HS state is reflected by the elongation of all Fe−N bonds by an average value of 0.215 Å (Figure 3a). What follows is the angular structural distortion of the entire **[Fe(1bpp)_2_]^2+^** cation which can be best described by considering three angles (Figure 3b): the α (bite) angle of 1bpp ligand, the dihedral θ angle between the planes of the two ligands, and the φ angle representing the rotation of one ligand with respect to the other about the iron atom. While only a small difference is observed between θ_LS_ (89.40°) and θ_HS_ (89.94°), the changes in φ_LS_ (178.15°) and φ_HS_ (172.98°), and in α_LS_ (80.08°) and α_HS_ (73.42°) are significant. Combined with the changes in bond lengths, the angular distortion causes a decrease of the unit cell's *c* axis by 0.55 Å, an increase of *b* axis by 0.05 Å, and an increase of β angle by 2°, which in total gives 2.6 % (35 Å^3^) decrease of the unit cell volume as the temperature is lowered. As can be expected, more or less severe distortion of the octahedral ligand field is reflected in the compound's electronic structure (Figures 3c and 3d) where a typical degeneracy of *t_2g_* and *e_g_* orbitals is lifted in both states. In the more deformed high spin state, the projected density of states (PDOS) curves of most *d* orbitals exhibit multiple peaks in a wide energy range indicating their contribution to multiple molecular orbitals. Based on our calculations, there are about 0.73 electrons in the spin‐down *d_xz_* orbital (*t_2g_*) and 0.24 electrons in the spin‐down $d_{x^{2}-y^{2}}$ and $d_{z^{2}}$ orbitals (*e_g_*). The partial occupancy of *d* orbitals is likely due to low symmetry and hybridization with ligands. A simplified diagram of *d*‐orbital occupations is shown on the right of Figure 3c to aid qualitative understanding. In comparison, PDOS curves for less deformed low‐spin state (Figure 3d) show one major peak for each *d* orbital clearly due to improved symmetry. In this case, there are about 0.90 electrons in the spin‐down *d_xz_* orbital. Overall, two spin‐up *e_g_* electrons turn into two spin‐down *t_2g_* electrons during a HS‐LS transition.

The above detailed analysis of the cation's geometrical parameters in HS and LS states not only helps to depict the structural changes related to the SCO transition but also implies an abrupt change of the cation's electric dipole moment when switching from one state to the other. This, in turn, makes **[Fe(1bpp)_2_](BF_4_)_2_** a promising candidate to show magnetoelectric coupling via SCO where the electric polarization can be sensed or controlled by a magnetic field and/or the magnetic properties by an electric field. More importantly, compared to the previously‐reported SCO materials showing the coupling,[^11^, ^12^, ^13^, ^14^, ^15^] **[Fe(1bpp)_2_](BF_4_)_2_** is particularly appealing because of its near‐room‐temperature SCO transition.

**Figure 4:**
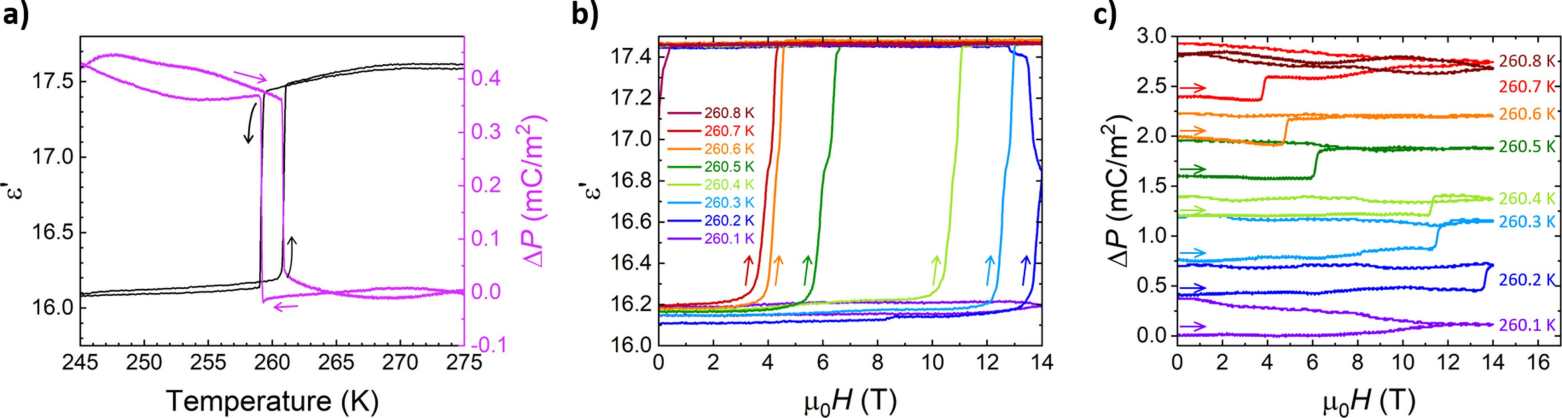
a) Temperature dependence of dielectric constant ϵ’ (left axis, black) and electric polarization Δ*P* (right axis, purple) collected along *b*‐axis direction with *T* sweep rate of 1 K min^−1^. Change in dielectric constant (b) and electric polarization (c) induced by magnetic field (μ_0_ *H*) of up to 14 T applied to [Fe(1bpp)_2_](BF_4_)_2_ for different temperature as indicated. Offset in c): 0.4 mC m^−2^.

To confirm whether the SCO transition is indeed electrically active, we first investigated temperature‐dependent electrical properties of the crystals. We collected the data on crystals pre‐cooled to 250 K and warmed back up to RT so that any strain related to the SCO is released prior to the application of the electrodes (see Figures S1 and S2). As shown in Figure 4a, the SCO transition reveals itself as an abrupt drop in the dielectric constant (ϵ′) value, measured along *b* axis, at 259 K on cooling and a sudden increase of ϵ′ at 260.5 K on heating which confirms the presence of ≈2 K hysteresis and a first‐order nature of the phase transition. We observed similar abrupt changes when collecting electric polarization (*P*) along *b* axis, which confirms the immediate change of the electric dipole moment at the SCO. The onset of Δ*P* occurs at the same temperature points on both cooling and heating as the onsets in the dielectric constant (Figure 4a).

Using the 2 K temperature hysteresis as a region of bistability of **[Fe(1bpp)_2_](BF_4_)_2_**, we further investigated the *H*‐induced switching of electrical properties from LS to HS state (Figures 4b and 4c) to demonstrate the magnetoelectric coupling. The data were taken with a history of slowly warming the crystal from 240 K to the measurement *T* in a 14 T superconducting magnet. 50 Oe s^−1^ sweep rate was used for ϵ′(*H*) and Δ*P*(*H*) collection. Starting from the dielectric constant (Figure 4b), the ϵ′(*H*) curves collected at the lowest (260.1 K) and the highest (260.8 K) temperature points correspond to the LS and HS states, respectively, and define two very well distinguishable levels of the dielectric constant: ≈16.15 for ϵ′_LS_ and ≈17.45 for ϵ′_HS_ (*conf*. Figure 4a). For the *T* points in the immediate vicinity of the SCO transition (*T*_c_), a change in the upsweep curves reaching ϵ′_HS_ value is clearly visible indicating that the sample switches from LS to HS state. The change is induced with the magnetic field as low as 3 T. Once switched by the applied magnetic field, the sample remains in the HS state, even in the absence of the field, as long as the temperature remains in the bistability region. Notably, based on the ϵ′(*H*) curve collected at 260.2 K, the progression of the transition does not stop when the magnetic field is being removed. Rather, the HS fraction grows until the whole crystal transition to the HS state. This dynamic is consistent with the one observed in the studies of nucleation and growth phenomena of SCO in **[Fe(1bpp)_2_](BF_4_)_2_** for the light‐induced transition where despite a small volume of the crystal being irradiated the whole crystal transformed progressively into the HS state presumably through long‐range elastic interactions.^[38]^

A field‐induced jump is also observed in the electric polarization Δ*P* (Figure 4c) for the same sample and for the same *T* points. The 0.3–0.4 mC m^−2^ change in Δ*P* values occurs while applying similar magnetic field as for ϵ′(*H*). While the field‐induced changes of ϵ′ and Δ*P* occur in a similar temperature window as the ones observed for *M*(*H*) (Figure 2b), a discrepancy in the rate of the transition progression between magnetic and electrical properties is noticeable. This might stem from additional SCO nucleation sites provided by electrodes (silver paint) applied on the opposite faces of the crystal used in electrical measurements.

The observed Δ*P*(*H*) at the SCO is of a similar order of magnitude as in previously reported magnetoelectric materials, Mn(taa),^[11]^ [Mn(3,5‐diBr‐sal)_2_323]BPh_4_,^[13]^ [Mn(3,5‐diCl‐sal)_2_323]BPh_4_,^[14]^ which confirms the high strength of magnetoelectric coupling achieved via spin crossover transitions, this time in Fe^2+^‐based material. As noted before, the origin of Δ*P* is the structural transition that significantly deforms the molecule in the polar *P*2_1_ space group setting. Based on these structural changes and different spin states (S=0 and S=2) of **[Fe(1bpp)_2_](BF_4_)_2_**, we have calculated theoretical Δ*P* by a density functional approach using the reported crystal structures at 240 K (LS) and 290 K (HS) as starting points. As shown in Table 1, the change in polarization, Δ*P*_tot_=−33.119 mC m^−2^, occurs mainly along the *b* crystal axis with the dominating ionic contribution Δ*P*_ion_=−98.932 mC m^−2^ and with the opposite in sign electronic contribution, Δ*P*_ele_=65.813 mC m^−2^. The results in Table 1 remain the same with and without on‐site Coulomb correction for fixed geometry. Noticeably, the calculated Δ*P*_tot_ value is two orders of magnitude larger than the experimental value, which often occurs due to either domain formation in crystals or additional dielectric compensation of the electric polarization.

**Table 1:** Change in the electric polarization along the lattice vectors *a*, *b*, and *c* as temperature increases. The values are based on the atomistic structures which are partially relaxed, i.e. relaxing the BF_4_^−^ counterions only, by DFT.

	*a*	*b*	*c*
Δ*P*_ion_ (mC m^−2^)	−0.017	−98.932	−0.054
Δ*P*_ele_ (mC m^−2^)	0.016	65.813	0.052
Δ*P*_tot_ (mC m^−2^)	−0.001	−33.119	−0.002

## Conclusion

To summarize, we have demonstrated strong magnetoelectric coupling at 260 K in the SCO compound, **[Fe(1bpp)_2_](BF_4_)_2_**. The coupling is realized through the interplay of structural, magnetic, and electrical changes that allows the SCO transition to be driven by the remarkably low magnetic field of 3 T. In this light, **[Fe(1bpp)_2_](BF_4_)_2_** represents an important example showing that SCO transitions can be utilized to achieve strong magnetoelectric coupling regardless of the transition temperature—the feature that holds promise for potential applications. In **[Fe(1bpp)_2_](BF_4_)_2_**, the coupling is enhanced by a strong and thermally switchable distortion of the lattice caused by the Jahn–Teller effect. Compared to the already reported magnetoelectric compounds where the coupling was accompanied by symmetry breaking,[^11^, ^12^, ^13^, ^14^, ^15^] the distortion in **[Fe(1bpp)_2_](BF_4_)_2_** occurs between phases of the same polar space group *P*2_1_ (an isostructural transition) indicating that large atomic displacements changing the value of electric polarization in neighboring phases are sufficient for the observation of the coupling.

## Conflict of interest

The authors declare no conflict of interest.

1

## Supporting information

As a service to our authors and readers, this journal provides supporting information supplied by the authors. Such materials are peer reviewed and may be re‐organized for online delivery, but are not copy‐edited or typeset. Technical support issues arising from supporting information (other than missing files) should be addressed to the authors.

Supporting Information

## Data Availability

The data that support the findings of this study are available in the supplementary material of this article.
