# Supplementary material for: Near‐Room‐Temperature Magnetoelectric Coupling via Spin Crossover in an Iron(II) Complex
Source: Angew Chem Int Ed Engl. 2022 Nov 23;61(52):e202214335. doi: 10.1002/anie.202214335 (PMC10099592; doi:10.1002/anie.202214335)
Supplement: Supplementary file 1 — Supporting Information [file ANIE-61-0-s001.pdf]

Angewandte Chemie
Eine Zeitschrift der Gesellschaft Deutscher Chemiker

Supporting Information

Near-Room-Temperature Magnetoelectric Coupling via Spin Crossover in an Iron(II) Complex

M. Owczarek, M. Lee*, S. Liu, E. R. Blake, C. S. Taylor, G. A. Newman, J. C. Eckert, J. H. Leal, T. A. Semelsberger, H.-P. Cheng, W. Nie, V. S. Zapf**

SUPPORTING INFORMATION

Table of Contents

1. Synthesis S2
2. Calorimetry measurements S2
3. Optical microscopy S2
4. Magnetization measurements... S3
5. Electric properties measurements S4
6. Theoretical calculations S4
7. References.. S6

1. Synthesis

All starting materials were used as received: $Fe(BF_4)_2 \cdot 6H_2O$ (97%, Sigma Aldrich), 2,6-di(1-pyrazolyl)pyridine (abrrev. 1bpp; 98%, TCI). **[Fe(1bpp)$_2$](BF$_4$)$_2$** was synthesized by mixing $Fe(BF_4)_2 \cdot 6H_2O$ (0.080 g; 0.237 mmol) and 1bpp (0.100 g; 0.473 mmol) in acetone (20 ml) and allowing the mixture to stir at room temperature for 1h. The resulting dark orange solution was concentrated to a half of its volume and filtered into a 40 ml crystallizing dish. The dish was placed in an empty desiccator with a vacuum inlet open (no vacuum applied) allowing for a very slow evaporation of the solvent. Within 2–3 days, dark-yellow crystals appeared. The crystals were kept undisturbed until a desired size was reached. Unit cell parameters determined from single-crystal X-ray diffraction were consistent with previous reports.[1-2]

2. Calorimetry measurements

Differential scanning calorimetry (DSC) measurements were performed using a Netzsch 204 F1 Phoenix DSC with a Netzsch CC300 LN_2 cryostat in sealed aluminum pans. Sample mass: 10.48 mg; temperature range: 240–280 K; scan rate: 10 K/min.

DSC curves were collected on crystals grown from acetone solution at room temperature. Mass of the sample was 10.48 mg. As can be seen in Figure S1, the anomaly corresponding to the first cooling process is much broader than the ones observed in the subsequent thermal cycles. The estimated enthalpy values for first and second cooling are 15.3 kJ/mol and 13.7 kJ/mol, respectively. We found that the sample undergoes irreversible mechanical deformation (microcracking) on first cooling, which may contribute to the higher apparent enthalpy. Itis plausible that the details of the first cooling depend on the conditions used for the crystal growth. As has been already discussed in the literature describing **[Fe(1bpp)$_2$](BF$_4$)$_2$** properties,[3] the change in enthalpy on first cooling were found to vary when crystals were grown from different solvents (acetone/Et_2O, $MeNO_2$/Et_2O, and MeCN/Et_2O) or at temperatures below the SCO transition. Although not investigated in our studies, it might be therefore possible to adjust the crystal-growth conditions so that the changes in mechanical properties of the crystals caused by SCO are minimal.

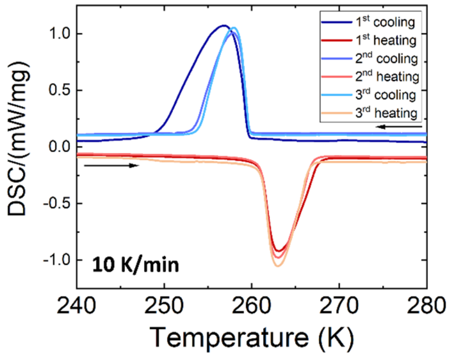

Figure S1. DSC curves obtained for **[Fe(1bpp)$_2$](BF$_4$)$_2$** crystals using 10 K/min temperature sweep.

3. Optical microscopy

Optical microscopy images of a **[Fe(1bpp)$_2$](BF$_4$)$_2$** single crystal were taken to analyze the changes in the quality of the crystal caused by the temperature-induced SCO transition. Although the images confirm that

the crystal stays intact through the three thermal cycles, substantial number of cracks appear after the first cooling/heating cycle (Figure S4), which explains the first cooling DSC data above. This suggests high lattice strain experienced by the crystal undergoing the transition due to the large change in lattice constants. Once released by the cracking process, the strain is much less detrimental for the crystal quality during the subsequent cycles.

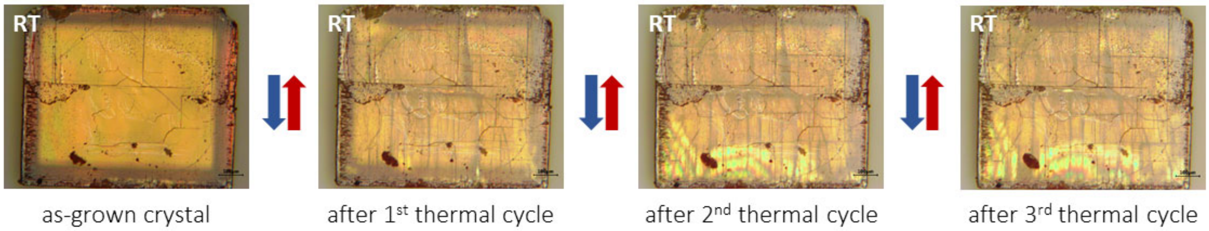

Figure S2. Optical microscopy images of a **[Fe(1bpp)$_2$](BF$_4$)$_2$** single crystal taken at room temperature (RT) after each of the subsequent cooling/heating cycle starting from the as-grown crystal. The scale in the photographs is 100 μm.

This observation helped us to modify the sample preparation for the collection of electrical data as the strain observed in the first thermal cycle strongly affected the quality of electrical contacts applied to the as-grown crystal, hence, preventing the collection of good quality data. To reduce the strain impact, the crystal was pre-cooled to 253 K in a refrigerator (under inert gas conditions to prevent water condensation on the crystals surfaces), and warmed back to RT. The contacts were then applied onto thin pieces of the crystal cut along *ac* crystallographic planes.

4. Magnetization measurements

The magnetization in a 13 T (Figure S2) and 14 T (Figure 2) superconducting (quasi-DC) magnets was measured using Vibrating Sample Magnetometers (VSM) in Physical Property Measurement Systems (PPMS) on a single-crystal of **[Fe(1bpp)$_2$](BF$_4$)$_2$**. Temperature for the collection of *M*(*H*) curves was reached by heating the systems from 250 K to the target temperature by using 0.1–0.3 K/min rate without overshoot. *M* was recorded with a VSM averaging time of 12 s and 50 Oe/s (Figure 2b) or 20 Oe/s (Figure S2) field scan rate. Low spin *M*(*H*) curves collected at 260.40 K (for the data in Figure 2) and 260.00 K (for the data in Figure S2) were used as a nonmagnetic background and were subtracted from the *M*(*H*) curves at other temperatures.

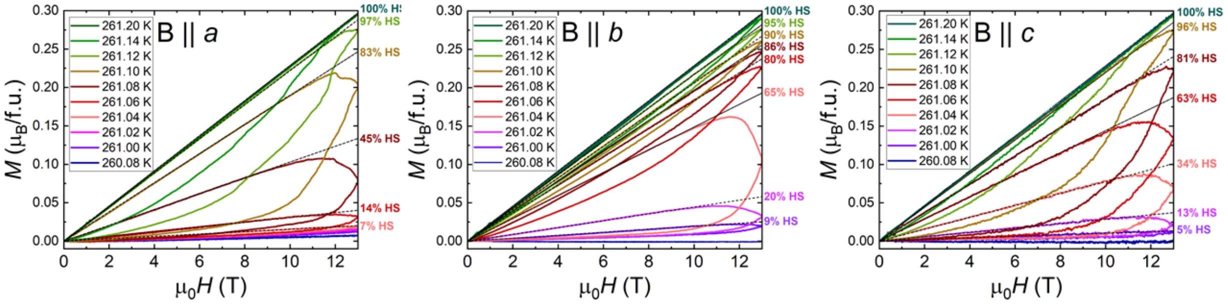

Figure S3. *M*(*H*) curves obtained in a 13 T superconducting magnet for single crystals oriented with their *a*, *b*, or *c* axis parallel the direction of the magnetic field. The crystals were attached to a quartz rod using GE varnish. 20 Oe/s sweep was used. The discrepancy in temperature between Figure 2b and Figure S2 comes presumably from the difference in thermometer calibration.

5. Electric properties measurements

A custom probe with Gore coaxial cables was used to measure dielectric constant and electric polarization. Crystals of **[Fe(1bpp)$_2$](BF$_4$)$_2$** (pre-cooled first to 253 K and warmed up to room temperature) were cut into thin slices along *ac*-plane using a razor blade. Silver paint contacts were applied to the parallel faces along the *b* axis. The size of the sample was approx. 0.5 × 0.3 × 0.15 mm. The temperature of measurements in ε'(H) and ΔP(H) was approached from 240 K using 0.1–1 K/min rate to avoid overshoot. The dielectric constant was determined by measuring the capacitance across the contacts with a Keysight E4980A Precision LCR meter at 100 kHz (no frequency dependence observed). ΔP(T) and ΔP(H) were measured by recording a change in surface charge on the contacts with a Keithley 6517A electrometer. A 14 T superconducting magnet was used for ε'(H) and ΔP(H) data collection; magnetic sweep rate used was 50 Oe/s; temperature sweep rate for ε'(T) and ΔP(T) was 1 K/min.

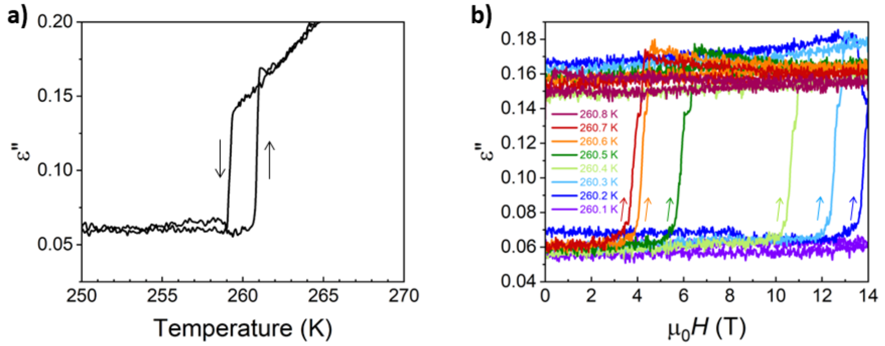

Figure S4. The imaginary part of the complex electric permittivity measured as a function of temperature (a) and magnetic field (b).

6. Theoretical calculations

We performed first-principles calculations based on density functional theory (DFT)[4-5] as implemented in the Vienna Ab initio Simulation Package (VASP)[6-7]. In our calculations, we adopted an energy cutoff of 600 eV for plane waves, the Perdew–Burke–Ernzerhof exchange-correlation energy functional[8], and the PAW pseudopotentials[9]. We sampled the reciprocal space by a 3×3×2 Monkhorst-Pack k-point grid[10] for structural relaxation and a 9×9×5 *k*-point grid for calculations of electric polarization[11-12]. During structural relaxation, van der Waals correction was included via the DFT-D3 method[13] and the lattice constants were fixed to the experimental values.[1] The energy and force tolerances were set to 10^{-8} eV and 0.001 eV/Å respectively. We applied the DFT+U method to calculate projected density of states (PDOS) with an on-site Coulomb parameter U = 5 eV and an exchange parameter J = 1 eV for Fe *d* orbitals. The DFT+U method was not applied for structural relaxations. In detail, PDOS analysis is based on calculations of isolated **[Fe(1bpp)$_2$](BF$_4$)$_2$** molecules. The atomic structures were fixed at the experimentally determined positions. The low-spin (high-spin) state is based on the structure at 240 K (290 K). We used a uniform background charge to account for the effects of counterions. According to our single-molecule calculations, the low-spin state is 0.78 eV lower in energy than the high-spin state (using DFT+U method). In the crystal environment, the energy difference becomes 0.82 eV per molecule. In principle, this difference in static

energy needs to be canceled by the entropy term of free energy to allow spin-crossover transition. The results of electric polarization calculations are based on atomistic structures in which the atoms of **[Fe(1bpp)$_2$]$^{2+}$** molecules are fixed at experimentally determined positions but the BF_4^- counterions are relaxed by DFT. If we also relax the **[Fe(1bpp)$_2$]$^{2+}$** cations, our calculations yield significant changes along the *a* and *c* crystal axes. This is, however, not observed in the experiments. It indicates that the structures fully relaxed by DFT are not reliable for this system. Calculations of bulk polarization may suffer from an artificial change in polarization by multiples of a polarization quantum[14]. We made sure that our results are free from such an artifact by enforcing continuous change in polarization for continuously changing structures (Figure S5).

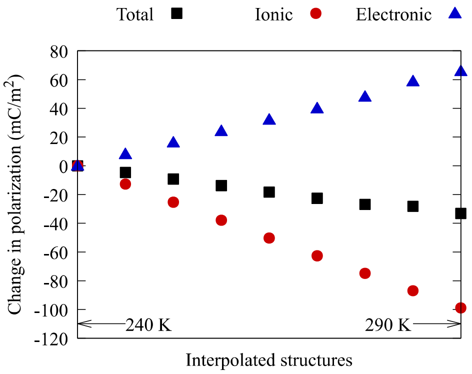

Figure S5. Change in calculated electric polarization as the atomistic structure changes. Linear interpolation between the structure at 240 K (LS) and the structure at 290 K (HS) is applied.

Figure S6a shows the crystal structure of **[Fe(1bpp)$_2$](BF$_4$)$_2$**, where Fe–N bonds not are aligned with the crystal axes. For the ease of analyzing electronic structure, we rotated the molecule until the Fe–N bonds are maximally aligned with the principal axes (see Figure S6b). The only Fe atom within a molecule is bonded with six nitrogen atoms which provide a deformed octahedral ligand field. The degree of deformation of the octahedral ligand field depends on the spin state. The deformation in the high-spin state is worse than that in the low-spin state as shown in Figure S6c.

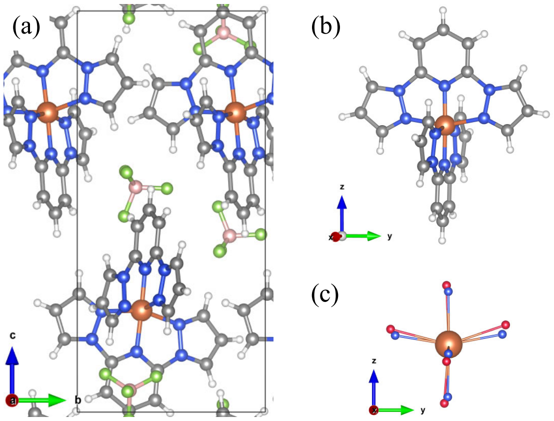

Figure S6: (a) A unit cell of the **$[Fe(1bpp)_2](BF_4)_2$** molecular crystal is marked by the solid lines. (b) A single **$[Fe(1bpp)_2]^{2+}$** cation in the low spin state. (c) Comparison of Fe–N bonds before (blue) and after (red) the spin crossover transition. Color scheme: brown, Fe; blue, N; gray, C; white, H; pink, B; green F.

7. References

[1] J. M. Holland, J. A. McAllister, Z. Lu, C. A. Kilner, M. Thornton-Pett, M. A. Halcrow, *Chem. Commun.* **2001**, 577-578.

[2] V. A. Money, I. Radosavljevic Evans, M. A. Halcrow, A. E. Goeta, J. A. K. Howard, *Chem. Commun.* **2003**, 158-159.

[3] C. A. Tovee, C. A. Kilner, J. A. Thomas, M. A. Halcrow, *CrystEngComm* **2009**, *11*, 2069-2077.

[4] P. Hohenberg, W. Kohn, *Phys. Rev.* **1964**, *136*, B864-B871.

[5] W. Kohn, L. J. Sham, *Phys. Rev.* **1965**, *140*, A1133-A1138.

[6] G. Kresse, *J. Non-Cryst. Solids* **1995**, *192-193*, 222-229.

[7] G. Kresse, D. Joubert, *Phys. Rev. B* **1999**, *59*, 1758-1775.

[8] J. P. Perdew, K. Burke, M. Ernzerhof, *Phys. Rev. Lett.* **1996**, *77*, 3865-3868.

[9] P. E. Blöchl, *Phys. Rev. B* **1994**, *50*, 17953-17979.

[10] H. J. Monkhorst, J. D. Pack, *Phys. Rev. B* **1976**, *13*, 5188-5192.

[11] R. D. King-Smith, D. Vanderbilt, *Phys. Rev. B* **1993**, *47*, 1651-1654.

[12] R. Resta, *Ferroelectrics* **1992**, *136*, 51-55.

[13] S. Grimme, J. Antony, S. Ehrlich, H. Krieg, *J. Chem. Phys.* **2010**, *132*, 154104.

[14] N. A. Spaldin, *J.Solid State Chem.* **2012**, *195*, 2-10.
